# Provider report cards as a scalable tool for outpatient antibiotic stewardship: insights from a medicaid claims-based approach

**DOI:** 10.1017/ash.2026.10752

**Published:** 2026-06-22

**Authors:** Mariana M. Lanata, Jacob T. Kilgore, Brandi Holthaus, Jonathan M. Willis, Tess Anderson, Caroline B. Samples, Jennifer Sparks, Joseph E. Evans, Michael J. Smith

**Affiliations:** 1 Department of Pediatrics, Division of Pediatric Infectious Diseases, Virginia Commonwealth University School of Medicine, Richmond, VA, USA; 2 Department of Pediatrics, Division of Pediatric Infectious Diseases, Marshall University Joan C. Edwards School of Medicine, Huntington, WV, USA; 3 Department of Pediatrics, Division of Pediatric Infectious Diseases, Duke University, Durham, NC, USA; 4 Department of Information Technology, Marshall University Joan C. Edwards School of Medicine, Huntington, WV, USA; 5 Marshall Health Network, Huntington, WV, USA; 6 Marshall University School of Pharmacy, Huntington, WV, USA

## Abstract

Antibiotic overprescribing remains common in pediatric outpatient care. We evaluated Medicaid claims–based provider report cards as a scalable stewardship tool. Quarterly audit and feedback improved guideline concordance, diagnostic appropriateness, and reduced cefdinir use; highlighting claims-based electronic feedback as a sustainable outpatient antimicrobial stewardship strategy suitable for statewide implementation.

## Introduction

Antibiotic overprescribing remains a major public health threat. In pediatrics, most antibiotic prescriptions occur in the outpatient setting, with approximately 50 million prescriptions written annually in the United States, and up to half deemed inappropriate.^1,2^ Although the Joint Commission implemented outpatient antimicrobial stewardship program (ASP) requirements in 2020,^3^ few pediatric institutions had established outpatient ASP infrastructure or dedicated support.^4^ Traditionally used ASP interventions, such as prospective audit and feedback, are challenging to implement broadly in the outpatient setting due to limited access and insufficient personnel.

West Virginia (WV) is consistently among the highest outpatient antibiotic-prescribing states in the United States, with the highest prescribing regions being rural and falling outside of the catchment areas of the few academic centers in the state.^8^ Insurance claims data provide an opportunity to support broader outpatient ASP efforts beyond a single institution/practice, but have primarily been used for retrospective analyses due to delays in data availability.^5–7^ In prior work, our group demonstrated the accuracy and validity of claims data for stewardship metrics.^9^ Building on this foundation and leveraging our unique access to near real time Medicaid claims (MC) data, this study evaluated provider report cards as a scalable outpatient ASP intervention and assessed their impact on targeted stewardship metrics in a pilot practice.

## Methods

### Study design

We conducted a before-after (prepost) study evaluating the impact of quarterly provider-specific prescribing feedback on prescribing habits in a pilot practice.

### Setting, patient population and access to data

Marshall Health Network (MHN) is a multisite academic network based in Huntington, WV. Prescriptions for pediatric patients from 0 to 19 years of age receiving care within MHN and insured by WV Medicaid were included. We targeted family medicine practitioners (MDs, DOs, and advanced practice providers [APPs]) and pediatricians (MDs and Dos) who prescribed antibiotics to pediatric patients within MHN. Trainees were excluded. Medicaid beneficiary eligibility, provider enrollment, pharmacy and medical claims data were provided by the West Virginia Bureau for Medical Services via a data use agreement. Data were received weekly via secure file transfer protocol (SFTP) and represented claims processed up to approximately 2 weeks prior. This study was approved by Marshall University Institutional Review Board.

### Metrics and interventions

**Figure 1.**
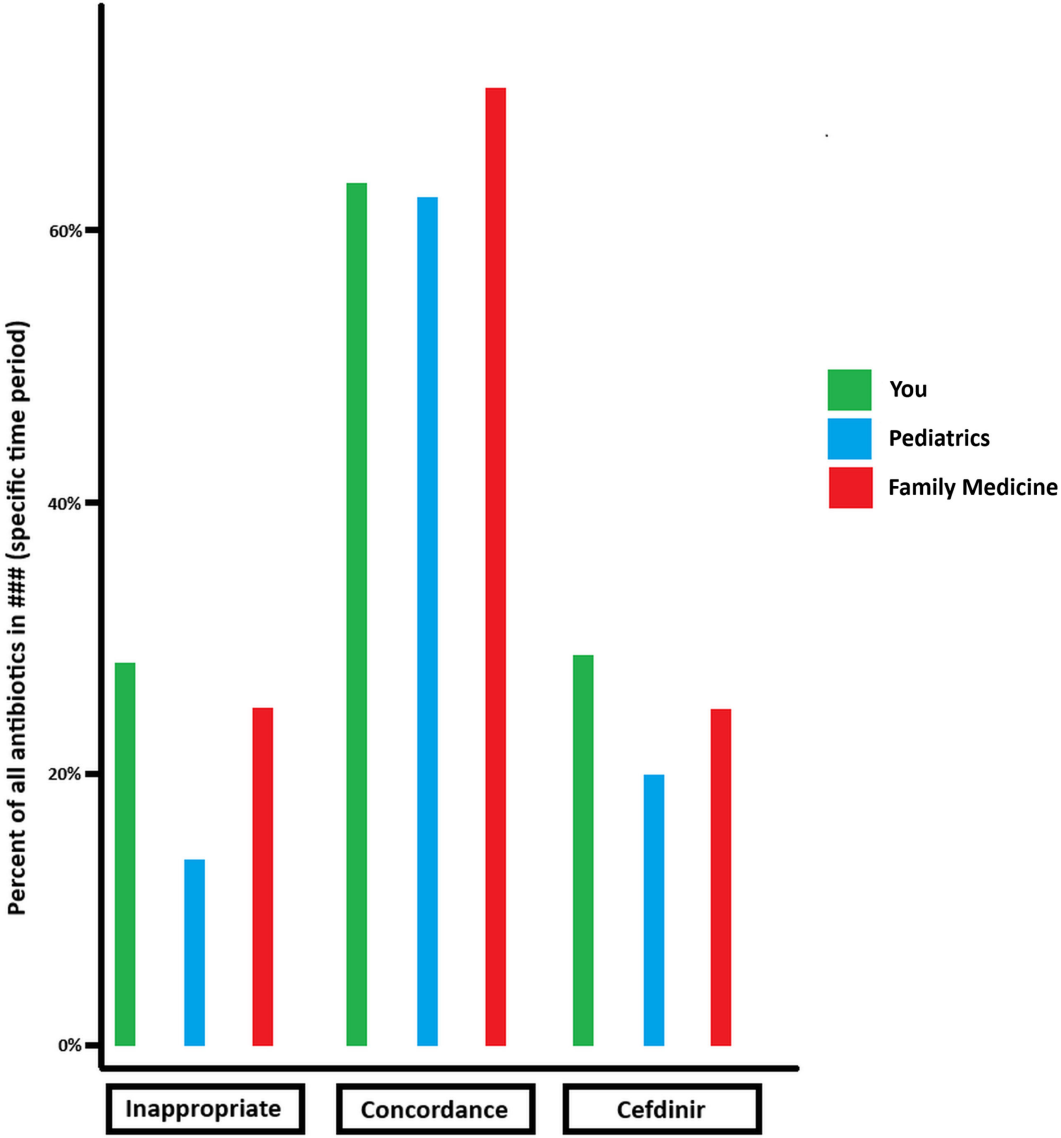
Figure 1 long description. Example of graph received by providers in their report card.

Using National Provider Identifier (NPI) numbers, we identified outpatient pediatric MCs from July 2021 – June 2022 (baseline) and July 2023 – June 2024 (postintervention). Dental claims and non-oral antibiotics (IV, IM, and topicals) were excluded. Using a previously published mutually exclusive scheme, diagnoses with antimicrobial prescriptions were classified as appropriate, potentially appropriate, and inappropriate.^10^ We developed provider report cards that assessed three key areas of focus (Figure 1).

1. ***Inappropriate prescriptions***: this represents diagnoses for which antibiotics are not indicated such as acute upper respiratory tract viral infections.
2. ***Guideline concordance:*** first line therapy in accordance with AAP/IDSA guidelines for acute otitis media (AOM), acute sinusitis (AS), acute pharyngitis (AP), community-acquired pneumonia (CAP), skin and soft tissue infections (SSTI), and urinary tract infections (UTI).
3. ***Cefdinir utilization***: the proportion of all prescriptions that were for cefdinir. This metric was selected due to previously identified overuse in WV despite its broad spectrum and the lack of first-line indications for common pediatric infections.^8^

Each provider’s performance was benchmarked against peers and communicated quarterly via individualized email reports. Prior to distributing the official reports, a small subset of providers was surveyed to obtain feedback and ensure that the reports were clear, user-friendly, and easily interpretable. One initial educational session was conducted for clinicians in the pilot practices prior to report dissemination to increase awareness, engagement, and understanding of the reports. In addition, each report included explanatory text with background information and links to relevant educational resources (Supplementary material 1).

### Statistical analysis

Provider-level prescribing outcomes were evaluated using mixed-effects grouped-binomial logistic regression models comparing pre-and postintervention prescribing performance while accounting for provider clustering through random-intercept effects. Separate models were constructed for each stewardship metric using aggregated provider-period data. Sensitivity analyses excluding low-volume providers were performed to assess robustness. Analyses were conducted in R version 4.4.1 using the lme4 and glmmTMB packages.

## Results

Fifty-six providers had at least one prescription during both pre-and postintervention periods and were included in the analysis. There were a total of 1,556 prescriptions in the preintervention period, and 2,285 prescriptions in the postintervention period. Prescribing for appropriate diagnoses increased markedly and was accompanied by a decrease in potentially appropriate diagnoses, while a smaller increase in inappropriate prescribing was observed. Guideline-concordant prescribing improved significantly postintervention, alongside a meaningful reduction in cefdinir use (Table 1). A sensitivity analysis excluding providers with less than 10 prescriptions confirmed these findings.

**Table 1.** Summary of targeted metrics: prescribing patterns in pre-and postintervention periods Table 1 long description.

		Preintervention (*n*= 1556)	Postintervention (*n*= 2,285)	OR (95% CI)	*p* Value	Observed trend
Prescription diagnosis appropriateness, *n* (%)						
Appropriate		203 (13.05)	601 (26.30)	2.4 (2.17–2.64)	<.001	Increased
Potentially appropriate		1,291 (82.97)	1526 (66.78)	0.41 (0.37–0.44)	<.001	Decreased
Inappropriate		62 (3.98)	158 (6.91)	1.67 (1.41–1.96)	<.001	Increased
Guideline concordant prescriptions, *n* (%)		918 (59.00)	1550 (67.80)	1.48 (1.34–1.61)	<.001	Increased
Cefdinir prescriptions, *n* (%)		172 (11.05)	170 (7.44)	0.64 (0.54–0.74)	<.001	Decreased

## Discussion

The implementation of quarterly provider-specific emails was associated with improvements across all three targeted prescribing domains, with measurable changes observed within a relatively short time frame. There were no other identified concurrent outpatient ASP interventions in this time frame that could have influenced these results. In contrast to traditional antimicrobial stewardship interventions, which are often labor-intensive and require sustained individual engagement, this approach leverages routinely collected insurance claims data. For rural states such as WV, where provider shortages pose a persistent challenge, claims-based stewardship offers a key advantage: once analytic algorithms are developed, report generation can be largely automated as new data become available, enabling scalable and sustainable stewardship efforts.

Our intervention was very successful despite providing a single educational session to each group of providers before distributing the report cards. We did not track engagement, confirm whether reports were opened, or offer any incentives such as continuing education credits or financial stimulus. The improvements in prescribing habits occurred without ongoing educational reinforcement during the study period, which may have implications for long-term sustainability.

Given the relatively short intervention period, it remains uncertain whether the observed changes will be sustained over time. In addition, our success may have been influenced by the setting of a small, close-knit healthcare network, where many primary care providers were familiar with the research team and had established trust in the intervention, potentially facilitating uptake of the feedback reports. As such, the impact of this approach may differ in larger or less interconnected healthcare systems. Changes in appropriateness metrics may also have been influenced in part by diagnostic coding shifts rather than prescribing behavior alone. Finally, the observed increase in inappropriate prescribing suggests that further refinement of feedback content and more targeted stewardship interventions may be necessary.

Building on these promising results, our team is expanding this work to develop a statewide antimicrobial stewardship dashboard and distribute provider report cards across WV through a strategic partnership with the WV Department of Health and Human Resources. This collaboration supports ongoing implementation while enabling targeted local interventions and in-person education in areas of greatest need, thereby enhancing sustainability, but also as a key strategy to anticipated further barriers to success as we expand efforts. We hope this framework can serve as a model for engaging other insurers to share routinely collected claims data, enabling a more comprehensive, population-level view of outpatient antibiotic prescribing across the state beyond a single insurer.

This study demonstrates the potential impact of provider-specific audit and feedback on outpatient prescribing and underscores the value of claims-based, real-time electronic feedback as a sustainable antimicrobial stewardship strategy suitable for statewide implementation.

## Supporting information

10.1017/ash.2026.10752.sm001 Lanata et al. supplementary material 1 Lanata et al. supplementary material

10.1017/ash.2026.10752.sm002 Lanata et al. supplementary material 2 Lanata et al. supplementary material

## Supplementary material

The supplementary material for this article can be found at https://doi.org/10.1017/ash.2026.10752.

## Figure 1. Long description

The bar graph compares the percentage of all antibiotics in three categories: Inappropriate, Concordance, and Cefdinir, among three groups: You, Pediatrics, and Family Medicine. The x-axis represents the categories, while the y-axis shows the percentage of all antibiotics in a specific time period. The graph features three vertical bars for each category, colored green for You, blue for Pediatrics, and red for Family Medicine. In the Inappropriate category, the green bar is slightly above 20 percent, the blue bar is around 10 percent, and the red bar is around 20 percent. In the Concordance category, the green bar is slightly above 60 percent, the blue bar is slightly below 60 percent, and the red bar is slightly above 60 percent. In the Cefdinir category, the green bar is around 30 percent, the blue bar is around 20 percent, and the red bar is around 30 percent. The graph highlights the differences in antibiotic prescribing practices among the three groups. All values are approximated.

Navigate back to Figure 1..

## Table 1. Long description

The table presents a comparison of prescription patterns between preintervention and postintervention periods. It includes data on prescription diagnosis appropriateness, guideline concordant prescriptions, and cefdinir prescriptions. The table has five rows and six columns, with column headers including Preintervention, Postintervention, OR (95% CI), p Value, and Observed trend. Row labels include Appropriate, Potentially appropriate, Inappropriate, Guideline concordant prescriptions, and Cefdinir prescriptions. Notable trends include an increase in appropriate prescriptions from 13.05 percentage to 26.30 percentage, a decrease in potentially appropriate prescriptions from 82.97 percentage to 66.78 percentage, and an increase in inappropriate prescriptions from 3.98 percentage to 6.91 percentage. Guideline concordant prescriptions increased from 59 percentage to 67.80 percentage, while cefdinir prescriptions decreased from 11.05 percentage to 7.44 percentage.

Navigate back to Table 1..
