# Supplementary material for: Provider report cards as a scalable tool for outpatient antibiotic stewardship: insights from a medicaid claims-based approach
Source: Antimicrob Steward Healthc Epidemiol. 2026 Jun 22;6(1):e184. doi: 10.1017/ash.2026.10752 (PMC13312243; doi:10.1017/ash.2026.10752)
Supplement: Lanata et al. supplementary material 1 — Lanata et al. supplementary material [file S2732494X26107529sup001.docx]

**TIDieR Checklist**

| **TIDieR Item** | **Description** |
| --- | --- |
| Brief Name | Quarterly provider-specific outpatient antibiotic prescribing report cards |
| Why | The intervention was designed as an audit-and-feedback antimicrobial stewardship strategy intended to improve outpatient antibiotic prescribing practices. We aimed to increase guideline concordance, reduce inappropriate prescribing, and decrease cefdinir utilization. |
| Materials | Providers received individualized electronic report cards containing prescribing metrics derived from near real-time Medicaid claims data, including inappropriate prescribing rates, guideline concordance metrics, and cefdinir prescribing rates. Reports also included peer comparison benchmarking. Recipients participated in an educational session prior to intervention rollout, and each report card was accompanied by a brief explanation of the metrics and additional stewardship educational resources. |
| Procedures | Medicaid claims data were extracted and analyzed quarterly. Prescribing metrics were calculated at the provider level using predefined stewardship definitions. Individualized report cards were generated and distributed electronically to participating providers. Reports were made using RStudio. |
| Who provided | The intervention was developed and implemented by a multidisciplinary antimicrobial stewardship team that included pediatric infectious diseases physicians, pharmacists and data analysts. |
| How | Report cards were distributed electronically via email. Since pilot practice was withing Marshall Health Network, provider emails were readily accessible to research team. |
| Where | The intervention was conducted in pediatric and family medicine outpatient practices affiliated with Marshall Health Network in West Virginia. |
| When and how much | Report cards were distributed quarterly throughout the intervention period. |
| Tailoring | Reports were individualized to each provider and included provider-specific prescribing data benchmarked against peer performance. |
| Modifications | No modifications occurred during this study period |
| How well (planned) | The study team planned standardized quarterly report generation and dissemination to all participating providers. Intervention adherence or fidelity were not assessed. |
| How well (actual) | Quarterly reports were successfully distributed to participating clinicians throughout the intervention period without failed electronic delivery notifications. While successful delivery to provider inboxes was confirmed, direct confirmation of report opening or engagement was not available. |
