# Supplementary material for: Provider report cards as a scalable tool for outpatient antibiotic stewardship: insights from a medicaid claims-based approach
Source: Antimicrob Steward Healthc Epidemiol. 2026 Jun 22;6(1):e184. doi: 10.1017/ash.2026.10752 (PMC13312243; doi:10.1017/ash.2026.10752)

Dear Prescriber,

Antimicrobial resistance is a growing public health threat placing the achievements of modern medicine in jeopardy. Did you know that West Virginia has one of the highest rates of antibiotic prescribing in the nation? According to statistics, most antibiotic prescribing occurs in the outpatient setting. Unless antibiotic prescribing improvements are made, the population will remain at risk.

The CDC developed the [Core Elements](https://nam02.safelinks.protection.outlook.com/?url=https%3A%2F%2Furldefense.com%2Fv3%2F__https%3A%2Fwww.cdc.gov%2Fantibiotic-use%2Fcommunity%2Fpdfs%2F16_268900-A_CoreElementsOutpatient_508.pdf__%3B!!OToaGQ!5f-d7Q7X9_UkgT67--1we9nb8WxS1iszV_sE7l7rOWKYeS4fNPKW7XJXl78Z7_7cnjMeQw%24&data=04%7C01%7Clanatapiazzo%40marshall.edu%7C54ab5dd2c8a94d6b79a408d9ffd2df78%7C239ab2783bba4c78b41d8508a541e025%7C0%7C0%7C637822103437625430%7CUnknown%7CTWFpbGZsb3d8eyJWIjoiMC4wLjAwMDAiLCJQIjoiV2luMzIiLCJBTiI6Ik1haWwiLCJXVCI6Mn0%3D%7C3000&sdata=disnJyt00ZExt2lTlOLeoKdtIqqh0KqsPoEAixYrpGg%3D&reserved=0) of Outpatient Antibiotic Stewardship to provide guidance in the battle against resistance. Tracking and reporting of antibiotic use is one of the recommended elements. We understand that individual providers and practices might not have the resources to do this on their own, so we would like to assist you in this important stewardship movement. A collaboration among antibiotic stewardship clinicians, Department for Medicaid Services, and the Department for Public Health has been established to assist West Virginia's fight against this threat.

WV Antibiotic Awareness is a campaign to reduce the impact and spread of antibiotic resistance by providing educational resources to healthcare professionals and their patients throughout the state.

A report of your outpatient antibiotic prescribing for children insured by Medicaid data has been attached. This report represents 6 months of your prescription activity for the year 2022; future reports will represent your previous month prescription habits. The data in the report are provided for your educational benefit and will not be shared publicly or used for reimbursement purposes.

Reporting metrics include inappropriate diagnosis, guideline concordance, and cefdinir prescription rates. Inappropriate diagnosis describes patient encounters where antibiotics are not warranted but were still prescribed. Examples of these are diagnoses such as acute upper respiratory tract infection or acute bronchitis, which have viral etiologies. Guideline concordance addresses the antibiotic choice itself to see if it aligns with current guidelines for common outpatient pediatric diagnoses and management. Concordance calculated for acute otitis media, bacterial sinusitis, community acquired pneumonia, streptococcal pharyngitis and urinary tract infections. We are also sharing cefdinir prescription rates as we have identified cefdinir as a frequently prescribed antibiotic in our region, yet it is not considered first line therapy for most common outpatient infections/management scenarios.

We encourage you to utilize the data to evaluate current practices and potentially impact your future antibiotic prescribing over the next calendar year. For more information on how these metrics were calculated, please visit our website.

Visit the [WV Antimicrobial Awareness website](https://jcesom.marshall.edu/departments-divisions/pediatrics/wv-antimicrobial-awareness/) to access the following resources: Implementation Workbook, customizable commitment posters, patient education materials, CE opportunities, and more.

For questions or assistance, email wvabxawareness@gmail.com.

Thank you for your work and effort to improve antibiotic prescribing in West Virginia.


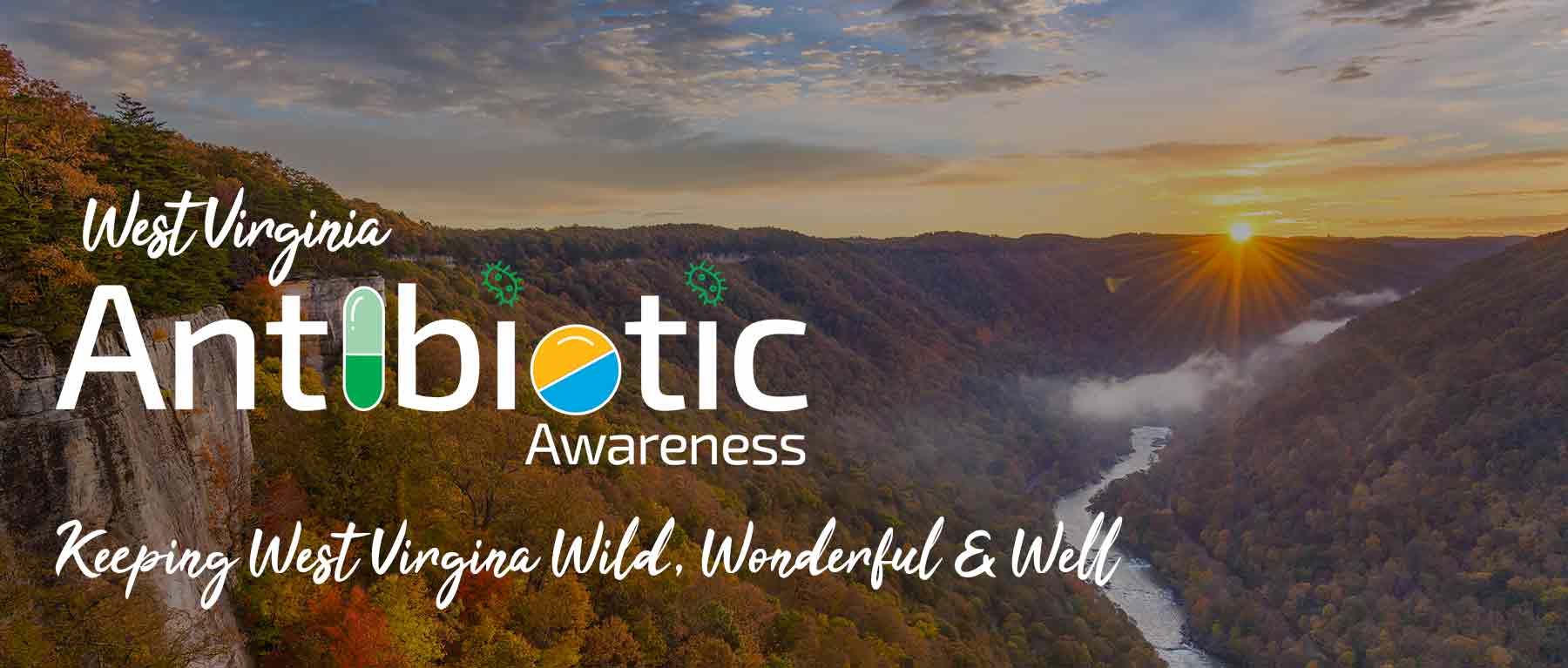

Supplement: Lanata et al. supplementary material 2 — Lanata et al. supplementary material [file S2732494X26107529sup002.docx]
